# Integration of Bayesian molecular clock methods and fossil-based soft bounds reveals early Cenozoic origin of African lacertid lizards

**DOI:** 10.1186/1471-2148-9-151

**Published:** 2009-07-01

**Authors:** Christy A Hipsley, Lin Himmelmann, Dirk Metzler, Johannes Müller

**Affiliations:** 1 Museum für Naturkunde – Leibniz-Institut für Evolutions- und Biodiversitätsforschung an der Humboldt-Universität zu Berlin, Invalidenstr. 43, 10115 Berlin, Germany; 2 Department of Ecology and Evolutionary Biology, University of California, Santa Cruz, A316 Earth and Marine Sciences Building, CA 95064, USA; 3 Institut für Informatik, Goethe-Universität, Robert Mayer Str. 11-15, D-60325 Frankfurt am Main, Germany; 4 Department of Biology, University of Munich (LMU), Grosshaderner Str. 2, D-82152 Planegg-Martinsried, Germany

## Abstract

**Background:**

Although current molecular clock methods offer greater flexibility in modelling evolutionary events, calibration of the clock with dates from the fossil record is still problematic for many groups. Here we implement several new approaches in molecular dating to estimate the evolutionary ages of Lacertidae, an Old World family of lizards with a poor fossil record and uncertain phylogeny. Four different models of rate variation are tested in a new program for Bayesian phylogenetic analysis called TreeTime, based on a combination of mitochondrial and nuclear gene sequences. We incorporate paleontological uncertainty into divergence estimates by expressing multiple calibration dates as a range of probabilistic distributions. We also test the reliability of our proposed calibrations by exploring effects of individual priors on posterior estimates.

**Results:**

According to the most reliable model, as indicated by Bayes factor comparison, modern lacertids arose shortly after the K/T transition and entered Africa about 45 million years ago, with the majority of their African radiation occurring in the Eocene and Oligocene. Our findings indicate much earlier origins for these clades than previously reported, and we discuss our results in light of paleogeographic trends during the Cenozoic.

**Conclusion:**

This study represents the first attempt to estimate evolutionary ages of a specific group of reptiles exhibiting uncertain phylogenetic relationships, molecular rate variation and a poor fossil record. Our results emphasize the sensitivity of molecular divergence dates to fossil calibrations, and support the use of combined molecular data sets and multiple, well-spaced dates from the fossil record as minimum node constraints. The bioinformatics program used here, TreeTime, is publicly available, and we recommend its use for molecular dating of taxa faced with similar challenges.

## Background

The molecular clock [1] has become an increasingly common tool among biologists for dating the origins of species or lineages using genetic sequence data. This is commonly done by measuring the genetic distance between two species and, assuming mutations occur at a constant rate, applying an external calibration to convert those distances into geological time (for a review of molecular clock methods, see [2]). Despite its widespread application, calibration of the clock using independent evidence, typically derived from the fossil record, is still problematic for many groups. While proper calibration dates for major evolutionary events like the mammal-bird or fish-tetrapod split are under constant debate [3-6], less attention has been given to smaller, less inclusive clades, which often have poor fossil records. Such discussions would be particularly useful for evolutionary biologists since it is often these clades that are the subject of more detailed investigations, e.g. in the context of biogeography or diversification. In the present study, we investigate how molecular divergences can be estimated in the absence of a good fossil record, and how fossil calibrations should be applied in such a case.

Here we combine a number of new approaches in molecular dating to assign evolutionary ages to the Old World lizard family Lacertidae (Squamata). Lacertidae, with about 280 species [7], is the dominant reptile group in Europe and a substantial component of the squamate reptile diversity in Africa. The family is divided into two subfamilies, the Gallotiinae and Lacertinae, with the latter group composed of two monophyletic clades, the mainly Palearctic Lacertini and Eremiadini of Africa (see [7] for a review of lacertid systematics). Compared to their Eurasian sister taxa, the African radiation shows extraordinary taxic diversity in desert habitats, while mesic-adapted genera in Africa are relatively species-poor. This disparity in species richness is surprising given that desert lacertids are considered evolutionarily younger and therefore have had less time to speciate than their mesic sister taxa, suggesting increased speciation rates in xeric habitat. Testing this hypothesis, however, has been difficult due to our lack of knowledge on the relative antiquity of desert clades. Terrestrial squamate fossils from the Cenozoic of Africa are rare [8], and this lack of fossil material has seriously hampered our ability to date the main lacertid lineages – a key step towards uncovering the ecological and evolutionary factors shaping their unique biogeographic patterns.

Based on previous molecular clock estimates, lacertids may have entered Africa (and at the same time split from the Palearctic clade) after Eurasia contacted Africa in the Neogene, some 17–19 million years ago (Mya) [7]. Fluctuating climatic conditions and aridification during that time may have promoted speciation in African lacertids through ecological displacement. According to Arnold [9], competitive interactions among species in mesic habitats forced subordinate taxa into drier, heterogeneous areas, resulting in niche divergence and diversification. However, a fundamental problem with this hypothesis is that other molecular divergence studies [e.g. [10,11]] have estimated a much older age for crown (and African) lacertids, pushing their origin far into the early Cenozoic. Furthermore, dates given by Arnold et al. [7] are largely based on Carranza et al. [12], which in contrast to other molecular clock studies relies on only a single calibration point for their estimates (the age of the Canary island El Hierro to calibrate the node between *Gallotia caesaris caesaris *and *G. c. gomerensis*). In addition, both of the above studies rely on the method of nonparametric rate smoothing [13] which may not properly account for rate variation as it has a tendency to overfit data, particularly for regions of the tree with short branches [14]. Therefore, thoroughly performed divergence estimates for Lacertidae, particularly for the African radiation, are still needed.

In this article, we estimate evolutionary relationships and divergence dates for the major lineages of Lacertidae with the goal of forming biogeographical hypotheses for their origin and subsequent spread throughout Africa. We construct a molecular phylogeny for the family using published nuclear and mitochondrial gene sequences, which for the first time are combined in a total evidence approach. We use multiple, well-spaced dates from fossil taxa within and outside of the family as independent calibrations. To account for uncertainty in paleontological dates we use flexible priors, meaning that calibrations are expressed as probabilistic distributions with minimum and maximum bounds [15,16]. The use of "soft" bounds is advantageous over simple point calibrations, as potential errors in fossil dating and identification, as well as the lag time between speciation and appearance of a fossil descendent, are statistically incorporated into the prior distribution [15-17]. Additionally, we test the reliability of our proposed calibrations by excluding individual priors and evaluating posterior estimates.

All molecular clock analyses are performed in a newly available software application for Bayesian analysis called TreeTime [18]. Like MrBayes [19] and BEAST [20], TreeTime uses a Metropolis-Coupled Markov Chain Monte Carlo (MCMCMC) method for Bayesian phylogenetic sampling. TreeTime simultaneously estimates tree topology and diversification dates and therefore does not require a starting tree topology, making it particularly appropriate for groups with uncertain phylogenies. Prior information on tree topology can be input by specifying two taxa, A and B, so that only trees in which at least one branch separates A from B are permitted. The user can also specify differently distributed priors for the time of the split between A and B. Within TreeTime, we implement four different models of rate variation and compare their performance using Bayes factor analysis. Phylogenetic relationships among the different genera are compared to previous studies, and age estimates from the different models are evaluated against available data from geology, climatology and the fossil record. Finally, we use our results as a platform for evaluating alternative hypotheses for the origins of Lacertidae and interpret our findings in light of paleogeographic trends in the Cenozoic.

## Methods

### Taxon sampling and alignment

Thirty-five species, representing 33 of 41 currently recognized genera, were used to construct a molecular phylogeny for the main lineages of Lacertidae. Partial DNA sequences of 3 mitochondrial genes (*12S*, *16S *and *Cytb*) and 2 nuclear genes (*Rag-1 *and *C-mos*) were retrieved from GenBank. Most lacertid genera are represented by a single species, with the exception of *Psammodromus *and *Mesalina*, which are each represented by two. All genes used in this study were not available for some of the species, so that six of the genera (*Acanthodactylus*, *Algyroides*, *Eremias*, *Nucras*, *Parvilacerta *and *Pedioplanis*) are represented by a combination of genes from two congeneric species. For example, the missing *Cytb *sequence of *Acanthodactylus boskianus *is substituted by that of *A. erythrurus*. Such substitutions at the genus level should have no effect on overall tree topology, since we are primarily interested in phylogenetic relationships of higher taxonomic units (i.e. above the generic level). The final data set for Lacertidae consists of 3 individuals from the subfamily Gallotinae (*Gallotia *+ *Psammodromus*), 15 individuals from Eremiadini corresponding to 14 genera, and 17 individuals from Lacertini each representing a single genus. Three additional species were used as outgroups: the teiid *Cnemidophorus tigris*, the amphisbaenian *Rhineura floridana*, and one of two living members of Rhynchocephalia, *Sphenodon punctatus*, as outgroup to all squamates. GenBank accession numbers for sequence data are listed in Table 1. Lacertid taxonomy follows Arnold et al. [7].

**Table 1:** GenBank accession numbers for mitochondrial and nuclear gene sequences used in the phylogenetic analysis of Lacertidae.

Species	*c-mos*	*rag1*	12*S*	16*S*	*cytb*
*Acanthodactylus boskianus*	EF632251	EF632206	AY633417	AY633441	AF206536^a^
*Adolfus jacksoni*	EF632253	EF632208	AF206615	AF206615	AF206539
*Algyroides fitzingeri*	EF632254^b^	EF632209^b^	AF206598	AF111177	AF206529
*Anatololacerta danfordi*	DQ461743	EF632224	AJ238188	AF080324	AF080323
*Apathya cappadocica*	EF632268	EF632223	AF145444	AF149946	AF080329
*Archaeolacerta bedriagae*	EF632256	EF632211	AF206592	AF206592	AF080326
*Cnemidophorus tigris*	AF039481	AY662620	AF206585	AY046492	AF006270
*Dalmatolacerta oxycephala*	EF632271	EF632228	AF440601	AF440616	AY256651
*Darevskia valentini*	EF632257	EF632212	AF206597	AF206597	LVU88611
*Dinarolacerta mosorensis*	EF632270	EF632227	AF440600	AF440615	AY151902
*Eremias arguta*	EF632258	EF632213	AY035827	AY035837	AF206549^c^
*Gallotia galloti*	EF632260	EF632215	AF206587	AF206587	AY151840
*Heliobolus spekii*	EF632262	EF632217	AF206608	AF206608	AF206544
*Hellenolacerta graeca*	EF632269	EF632225	AF440602	AF440617	AF080272
*Iberolacerta monticola*	EF632265	EF632220	AF440589	AF440604	AY151872
*Ichnotropis squamulosa*	EF632266	EF632221	AF080365	AF080367	AF080366
*Lacerta agilis*	EF632267	EF632222	AF149947	DQ494823	AF080299
*Latastia longicaudata*	EF632272	EF632229	AF206609	AF206609	AF206545
*Meroles suborbitalis*	EF632273	EF632230	AF206611	AF206611	AF206540
*Mesalina guttulata*	EF632274	EF632231	AY218019	AY217969	AY217815
*Mesalina rubropunctata*	EF632275	EF632232	AY035830	AY035840	EF555274
*Nucras tessellata*	EF632276^d^	EF632233^d^	AF206612	AF206612	AF206550
*Omanosaura jayakari*	EF632277	EF632234	AF080350	AF080352	AF080351
*Ophisops elegans*	EF632278	EF632235	AF206605	AF206605	AF206532
*Parvilacerta fraasii*	EF632279^e^	EF632236^e^	AJ238187	AF080318	AF080317
*Pedioplanis namaquensis*	EF632280^f^	EF632237^f^	AF206613	AF206613	AF206546
*Phoenicolacerta laevis*	DQ461740	EF632226	AJ238183	AF080333	AF080332
*Podarcis muralis*	EF632282	EF632239	AF206600	AF206600	AY151912
*Poromera fordii*	EF632283	EF632240	AF080368	AF080370	AF080369
*Psammodromus algirus*	EF632284	EF632241	AY218020	DQ298734	AY217816
*Psammodromus hispanicus*	EF632285	EF632242	DQ298606	DQ298676	DQ298562
*Rhineura floridana*	AY444021	AY662618	AY881097	AY605473	AY605473
*Sphenodon punctatus*	AF039483	AY662576	AF534390	DQ267621	AF534390
*Takydromus sexlineatus*	EF632288	EF632245	AF206589	AF206589	AY248472
*Tiera dugesii*	EF632289	EF632246	AF543309	AF080315	AF080314
*Timon lepidus*	EF632290	EF632247	AF206595	AF206595	AY151899
*Tropidosaura gularis*	EF632291	EF632248	AF206616	AF206616	AF206541
*Zootoca vivipara*	EF632292	EF632249	AF206594	AF206594	AY151913

^a ^*Acanthodactylus erythrurus*

^b ^*Algyroides moreoticus*

^c ^*Eremias velox*

^d ^*Nucras lalandii*

^e^*Parvilacerta parva*

^f^*Pedioplanis undata*

Species substitutions for missing gene sequences are noted by superscripts.

Alignments were performed separately for each gene using ClustalW [21] and manually corrected in SEAVIEW [22]. A total of 15–20 base pairs (bp) of *16S *that could not be aligned unambiguously were excluded from the analysis. Final gene lengths are 254 bp *16S*, 327 bp *12S*, 281 bp *Cytb*, 1012 bp *Rag-1 *and 375 bp *C-mos*. To test for incongruence among genes, a partition homogeneity test [23] was conducted in PAUP* 4.0b10 [24]. The test (100 replicates of random addition heuristic search option with tree-bisection-reconnection branch swapping) indicated significant heterogeneity among genes (p = 0.01). However, since a growing number of studies indicate that incongruence tests are not reliable indicators of data set combinability [25] and no strongly supported nodes were in conflict with previous studies, genes were concatenated into a multigene data set of 2249 bp. Following a total evidence approach [26], the following analyses were conducted on the combined data to maximize the amount of characters and explanatory power of the available data. As a test of our combined approach, we also analyzed partitioned mitochondrial DNA (mtDNA) and nuclear DNA (nDNA) sequences for one of the relaxed clock models (Uncorrelated lognormal with 10% prior probability distributions, described below). These values were then compared to results from the concatenated data set to explore possible biases associated with the different genomes.

### Phylogenetic and molecular clock analyses

Divergence dates for Lacertidae were estimated under four different Bayesian molecular clock models. Minimum constraints for five nodes were chosen based on evidence from the fossil record. In a conservative approach, the oldest age of the stratigraphic layer in which a fossil was found was used to represent the earliest occurrence of that lineage, and potential calibrations were limited to fossils that are reliably assigned to extant clades. Calibrated nodes are: (i) *Sphenodon punctatus *– *Cnemidophorus tigris*, 228.0 Mya, based on the earliest identified rhynchocephalian from the late Triassic [Carnian; [27]], and corroborated by the oldest-known fossil squamate, *Tikiguania*, from the Carnian of India [28], (ii) *Cnemidophorus tigris *– *Rhineura floridana*, 113.0 Mya, corresponding to the oldest known teiid, *Ptilotodon*, from the lower Cretaceous [Aptian-Albian; [29]], (iii) *Rhineura floridana *– *Gallotia galloti*, 64.2 Mya, based on the fossil rhineurid *Plesiorhineura *from the Paleocene [Torrejonian; [30]], and (iv) *Timon lepidus *– *Dalmatolacerta oxycephala*, 5.3 Mya based on the Pliocene "*Lacerta ruscinensis" *from Roussillon, France, whose fossil remains are indistinguishable from the modern *T. lepidus *presently living in the same area [31].

**Figure 1:**
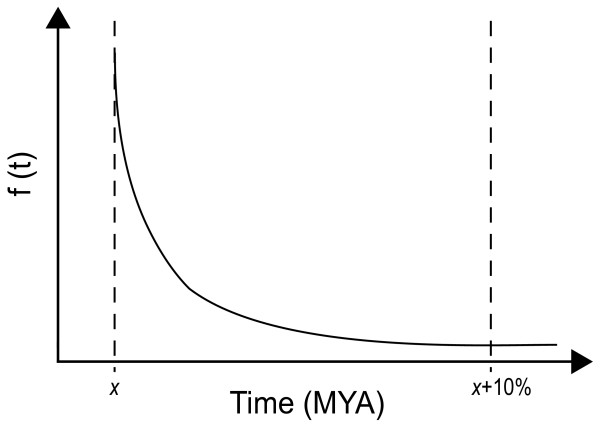
**Exponential prior probability distribution with a minimum bound**. The probability that the actual divergence date occurs earlier than the fossil calibration *x *declines according to an exponential distribution, with 95% of the posterior density within 10% of the fossil age.

To incorporate uncertainty surrounding fossil calibrations, prior constraints are expressed as probability based distributions. We use a rigid, or "hard", minimum bound, meaning that the true divergence date cannot be younger than the earliest known fossil. The probability that the divergence event occurred above the minimum date declines according to an exponential distribution, such that 95% of the posterior density falls within the range [x - x + 10%] (Figure 1). For example, the minimum age constraint for the split between Rhynchocephalia and Squamata is 228 Mya, and the expected posterior estimate is between 228.0 and 239.4. To test the sensitivity of posterior estimates to prior distributions, we also allow expectancy values for calibrated nodes to fall within 20% of the minimum age, so that 95% of the posterior density is between [x - x + 20%]. This allows us to evaluate the influence of the range of soft bounds used for a given data set, irrespective of possible errors in fossil calibration dates.

In addition to estimating divergence dates, we evaluate the reliability of our proposed fossil calibrations by systematically removing individual priors and comparing posterior estimates. Specifically, we test the accuracy of the dates proposed for amphisbaenians (*Plesiorhineura*, 64.2 Mya) and teiids (*Ptilotodon*, 113.0 Mya) using three different treatments. In the first treatment, both the amphisbaenian and teiid are excluded so that only the oldest date (Rhynchocephalia, 228 Mya) and youngest date (*Lacerta ruscinensis*, 5.3 Mya) remain. In the second and third treatments, only the amphisbaenian or teiid is removed, respectively. If a calibration is accurate, provided the remaining calibrations are reliable and the data and model are appropriate, the posterior estimate should remain within the prior range even in the absence of the fossil constraint. If the calibration is poor, the posterior should move away from the prior [32]. This approach also allows us to compare our results with other studies using similar combinations of fossil calibrations to date the origins of squamate groups [see [10,11]].

For nucleotide data, all models are nested in the General Time Reversible model of sequence evolution with a proportion of invariant sites and gamma distributed rate heterogeneity (GTR+I+Γ), as determined by jModelTest 0.1.1 [33,34]. For each analysis, the MCMC was run for 50,500,000 steps each chain and sampled every 500,000 steps. The first 1,000,000 steps of each run were discarded as burnin. To couple the four parallel chains we used a heating coefficient of 0.3. This resulted in a sample of size of 100 from the posterior distribution, taken from the cold chain.

MCMC calculations were performed in the program TreeTime, freely available at [18]. Within that program, the following models were implemented:

MC: Strict molecular clock model [1], assumes a fixed rate of evolution along all branches of the tree.

CPP: Compound Poisson Process [35], in which points of rate change are interspersed along branches. Following each substitution event, the current rate is modified according to a Poisson process with an adaptive intensity, which determines the *a priori *distribution of the number of changes. Rate modulations are gamma distributed, such that the expectancy value of the product of multiple rate changes is equal to 1.

ULN: Uncorrelated lognormal distributed model of Drummond et al. [16], in which the evolutionary rate of each branch is independently drawn from a lognormal distribution. There is no autocorrelation of rates between neighbouring branches. Parameters within the model determine the expectancy value and variance of rates. A smaller variance indicates a smaller deviation from the strict molecular clock, since rates of change are similar across branches.

DM: Dirichlet model [36]. The *a priori *distribution of evolutionary rates at the branches follows a dirichlet distribution. Parameters within the model determine the variance of rates. The smaller the variance, the smaller the deviation from a strict molecular clock. The average evolutionary rate across branches is kept constant, so that only relative differences between rates are considered.

As an independent evaluation of our results, we also calculate divergence dates for Lacertidae under the ULN model in BEAST [20], an alternative program for Bayesian analysis. Identical model parameters were used in the two programs with the following exceptions: 1) In addition to priors for calibrated nodes, BEAST requires a prior for the distribution of divergence dates, for which we chose the Yule process. 2) BEAST estimates the equilibrium distribution of nucleotides only once at the beginning of the analysis, TreeTime samples these estimates continuously. 3) In BEAST the molecular clock is relaxed by varying molecular rates of the substitution model among branches, for which reason the rates are dependent on the time scale of the tree. TreeTime compresses or stretches the lengths of branches in the tree, given in molecular time units, by rate multipliers with a mean of one.

Finally, we test the performance of alternative clock relaxations on our data by computing Bayes factors, a Bayesian alternative to likelihood ratio tests. Bayes factors calculate the ratio of marginal likelihoods between two given models by integrating over all possible parameter values (as opposed to estimating the maximum likelihood for each parameter). In a comparison between models M_1 _and M_2_, a Bayes factor >10 on a logarithmic scale indicates that M_1 _is more strongly supported by the data under consideration than M_2 _[37]. A significant advantage of Bayes factors over likelihood ratio tests is that they automatically penalize models with increasing complexity, and thus guard against overfitting. Furthermore, by using the strict molecular clock as a reference, they allow for a general comparison among any number of independent models [38].

## Results

**Figure 2:**
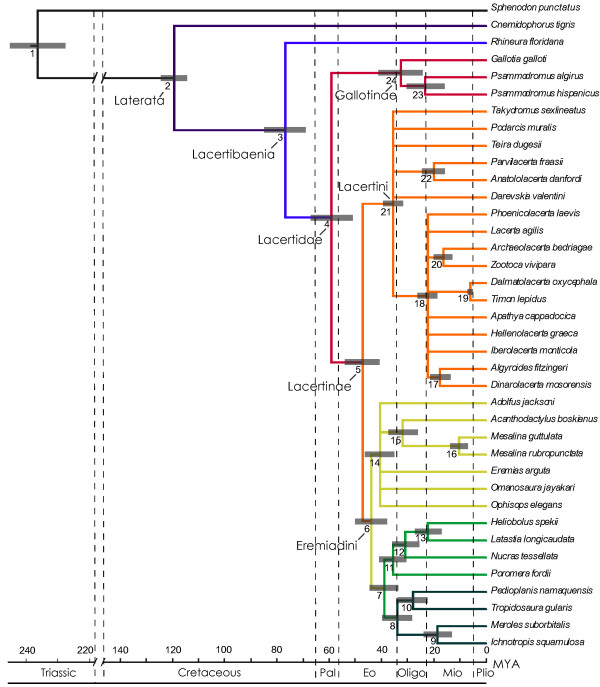
**95% majority rule consensus tree for Lacertidae with divergences estimated under an Uncorrelated Lognormal relaxed molecular clock, based on a concatenated data set of 3 mitochondrial and 2 nuclear genes**. Gray bars represent mean divergence dates ± 1 standard deviation. Nodes are numbered consecutively and correspond to node numbers in the Additional file 1. A geological time scale in millions of years is shown below.

Phylogenetic analysis of the combined genetic data recovers the major lineages of Lacertidae in accordance with previous studies [e.g. [7,39]]. The subfamily Gallotinae appears most basal and is sister to Lacertinae, which contains the subclades Lacertini and Eremiadini. In all cases the amphisbaenian *Rhineura floridana *forms the sister taxon to Lacertidae, as suggested by previous studies [10,40]. The four independent Bayesian analyses differed only slightly in their tree topology, so that only the tree with the highest posterior probability is shown here (ULN 95% consensus tree, Figure 2).

In all phylogenetic analyses, relationships within Gallotinae were identical, however some differences in tree topology exist regarding the subfamily Lacertinae. The Eurasian subclade Lacertini is represented by a comb-like topology, where generic relationships are largely unresolved. In contrast, the African radiation is split into two monophyletic groups corresponding to Saharo-Eurasian and Ethiopian (Africa south of the Sahara Desert) distributions [e.g. [7,39]], with the latter divided into southern and east African subclades. Phylogenetic resolution is generally higher among African genera, with the only differences between trees limited to the placement of *Acanthodactylus boskianus *as sister taxon to *Mesalina *in all cases except for the CPP 20% model, and *Poromera *as outgroup to the inclusive *Nucras*/*Latastia*/*Heliobolus *in all cases except for the strict molecular clock (MC), where it instead branches from the base of the Ethiopian clade. These slight variations in topology have no affect on relationships among the major lineages, so they are not discussed further here. Overall, all trees are essentially in agreement and in the following discussion we refer to the single tree shown in Figure 2.

### Divergence estimates

Divergence dates for Lacertidae estimated from each of the molecular clock analyses are listed in Additional file 1: **Lacertid clade ages**. For nearly all of the relaxed clock models, the origin of modern lacertids, as indicated by the split between Gallotinae and Lacertinae, is estimated to be in the Paleocene (56–58 Mya), with the initial radiation of the African clade occurring in the mid-Eocene (44–46 Mya). Within the Eremiadini, the separation of the Saharo-Eurasian and Ethiopian clades occurred after their split from the Lacertini, 40–43 Mya. The subfamily Gallotinae diverged into its component genera, *Gallotia *and *Psammodromus*, during the Oligocene, 29–32 Mya.

To assess the relative fitness of the alternative clock relaxations, we calculated Bayes factors between each model using the strict molecular clock as a reference. Results are shown in Table 2 on a logarithmic scale. In all comparisons, the strict molecular clock was strongly rejected in favour of relaxed clock models, with Bayes factors ranging from -170 to -101. Among the different clock relaxations, the CPP model performed most poorly and gave considerably younger ages for almost all nodes. The DM and ULN model received comparable Bayes factors, though ULN performs slightly better (ln ULN_DM = 24). Taken together, the relative ordering of MC, CPP, DM, and ULN indicates that the Uncorrelated lognormal model is most appropriate for our data set.

**Table 2:** Natural logarithm of Bayes factors for the molecular clock models Compound Poisson Process (CPP), Dirichlet Model (DM), Uncorrelated lognormal (ULN), and the strict Molecular Clock (MC), based on the concatenated data set.

	CPP	DM	MC	ULN
CPP		31	-101	56
DM	-31		-133	24
MC	101	133		157
ULN	-56	-24	-157	

In addition to our original divergences calculated with a 10% maximum soft bound, we expanded probability ranges to within 20% of the minimum date. Doubling prior bounds increased divergence estimates for all nodes, as well as widening confidence intervals (Additional file 1). For example, the original bounds for the Amphisbaenia-Lacertidae split were (64.2, 70.6) and the posterior estimate from to the ULN model was 68.5–83.3 Mya. When prior bounds were increased to 20%, the prior range became (64.2, 77) and the posterior estimate increased to 77.2–100.2 Mya (Additional file 1, node 3). The smallest changes resulting from this increase occurred at the *Sphenodon*-Squamata and Teiidae-Amphisbaenia nodes, which increased by an average of 3.2% and 7.5%, respectively. The largest change occurred at the *Timon*/*Dalmatolacerta *node, where the divergence date increased by an average of 43.6% across all models, more than double that of any other posterior expansion. Effects were most dramatic in the CPP model, which without exception produced the largest increase in divergence estimates and standard deviations when prior distributions were expanded to 20%. However, because the CPP model is unreliable for our data (see Bayes factors, Table 2), we ignore these dates in the final discussion.

**Figure 3:**
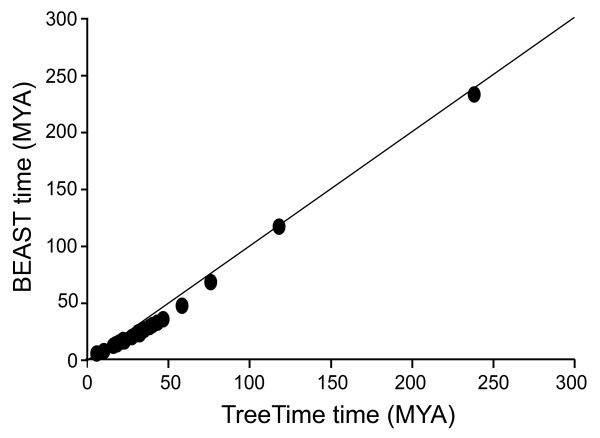
**Comparison of divergence dates estimated in the Bayesian programs TreeTime and BEAST**. Mean molecular divergence dates, in millions of years, estimated under the ULN relaxed molecular clock model with a 10% prior probability distribution in TreeTime plotted against dates estimated in BEAST for all nodes in the Additional file 1. The solid line indicates a 1:1 relationship between the two values.

The BEAST analysis of the combined data resulted in a tree topology identical to TreeTime, except for the loss of the *Archaeolacerta/Zootoca *sister group. Molecular dates were younger in BEAST for all but one node (Figure 3; Additional file 1, node 19), but still all fall within the 95% confidence intervals produced in TreeTime. The most significant differences occur at the major lacertid splits (Additional file 1, nodes 4, 5 and 6), where divergences occur approximately 10 My later. These changes are most likely attributed to differences in model parameters and not performance of the programs themselves. The prior distribution on branching times used in BEAST, the Yule Process, has a tendency to pull divergence dates towards the tips of the tree when basal internodes are short but terminal branches are long. This influence can be even stronger when rates vary inside the tree, as is most likely the case in Lacertidae. In TreeTime, prior information on branching times is applied only to calibrated nodes, and every allocation of branching times for remaining nodes in the tree is equally likely. Because of these differences in program settings, we refer only to the age estimates given by TreeTime for our discussion.

### Partitioned data sets

**Figure 4:**
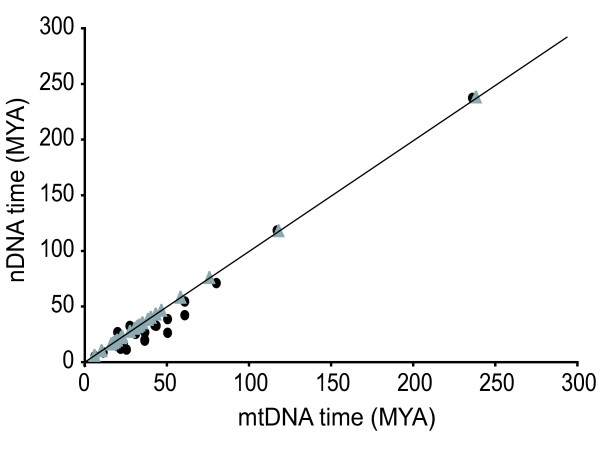
**Comparison of mitochondrial DNA and nuclear DNA based estimates of divergence times**. Mean molecular divergence dates, in millions of years, estimated from partitioned mtDNA and nDNA for selected nodes. Gray triangles show dates based on the combined data (mtDNA + nDNA), plotted against both axes. The solid line indicates a 1:1 relationship between mtDNA and nDNA estimates. All estimations were made under the ULN relaxed molecular clock model in the program TreeTime.

Phylogenies based on the partitioned mtDNA and nDNA do not reach a resolution equivalent to the combined data set, leaving large parts of Eremiadini and Lacertini unresolved. Although we refrained from constraining nodes prior to the analysis since there is no current consensus on lacertid ingroup phylogeny, all major nodes were still recovered. Overall, mtDNA produced older dates when compared to the nDNA and combined data. Node ages based on nDNA alone were marginally younger than in the combined analysis (Figure 4; Additional file 1). Among the major nodes, dates among the partitioned and combined data vary little, with the largest difference being the Amphisbaenia-Lacertidae split. Mean dates for the major clades, including European and African lacertids, still remain within the 95% confidence intervals of the combined data.

### Selective deletion of calibration points

**Figure 5:**
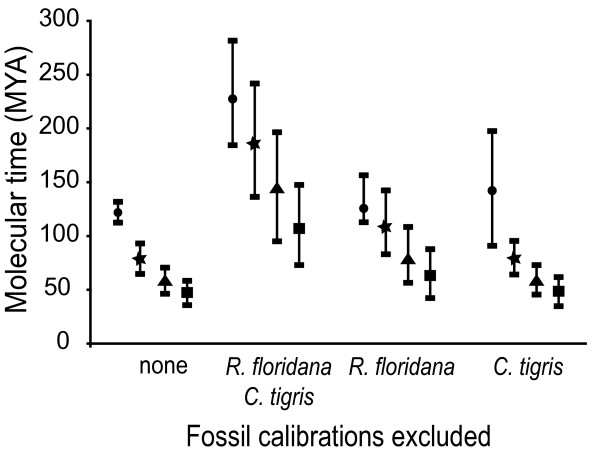
**Influences of individual calibration points on node ages**. Mean molecular divergence dates, ± 1 standard deviation, for the major lineages estimated using different combinations of fossil calibrations. Divergence dates were calculated using: all four fossil calibrations; all excluding the teiid *Cnemidophorus tigris *and the amphisbaenian *Rhineura floridana*; all except *R. floridana*; and all except *C. tigris*. Evolutionary splits are Teiidae-Amphisbaenia (circle), Amphisbaenia-Lacertidae (star), Gallotinae-Lacertinae (triangle), and Lacertini-Eremiadini (square).

Three analyses were run under the ULN model each excluding one or more fossil priors. In almost all cases, estimated divergence dates were older and had larger standard deviations than when all calibrations were used (Figure 5). The largest changes occurred when both the amphisbaenian and teiid calibrations were removed. With the exception of the most recent split (*Mesalina guttulata/Mesalina rubropunctata*), divergence estimates became significantly older and standard deviations expanded by 5–20 My. Excluding the amphisbaenian calibration caused posterior ranges to increase by up to 30 My. Age increases were most strongly evident at the origins of the major lineages. Removing the teiid calibration alone had the least effect on posterior estimates, with a maximum increase of 2 My at all nodes (except for the Teiidae-Amphisbaenia split itself, which increased by almost 15 My).

## Discussion

In general, our results confirm recent molecular based studies of lacertid phylogeny, including their sister relationship with amphisbaenians. Low taxon sampling within the subfamily Gallotinae hinders any phylogenetic interpretations for the group, apart from being a monophyletic clade that forms the outgroup to the remaining lacertid taxa. The Palearctic clade Lacertini forms a large polytomy in the strict consensus tree that includes the Asian grass lizard *Takydromus sexlineatus *(Figure 2). Relationships among the remaining Palearctic genera are poorly resolved and are therefore not discussed further here, though some biogeographical implications for the clade in general are considered below.

Several well supported sister groups occur within the African subclade Eremiadini (Figure 2). The Saharo-Eurasian group consists of the mainly North African/Asian genera *Ophisops*, *Acanthodactylus*, *Eremias *and *Mesalina*, as well as the Middle Eastern *Omanosaura *and equatorial African genus *Adolfus*. Within the Ethiopian clade, the taxon pairs *Pedioplanis*/*Tropidosaura *and *Meroles*/*Ichnotropis *form a distinct southern African cluster, while the remaining genera are distributed primarily in east Africa (with the exception of the singular species of *Poromera *in western central Africa). The restricted distribution of *Poromera *in equatorial forests may be the result of high levels of extinction some time in the past. Indeed, Africa's rainforests have diminished greatly since the early Cenozoic, and during the last 30 My a trend toward increasing aridity, coupled with repeated glacial phases, has left only small remnants of the once extensive blocks of rainforest [41].

### Divergence estimates for Lacertidae and their evolutionary implications

Mean dates for the origin of Squamata based on the DM and ULN model (236.9, 238.2 Mya) fall well within estimates given by Vidal and Hedges [10] (221–251 Mya) based on nine nuclear genes, two of which are included in the present study. The split between amphisbaenians and lacertids, on the other hand, is not as well supported. Although their sister relationship is corroborated under all models, node ages vary by over 10 My in the 10% and 20% analyses, the latter case placing the split almost 30 My earlier than the earliest known rhineurid. Previous studies by Vidal and Hedges [10] and Wiens et al. [11] give much older dates for amphisbaenians, pushing their origin back to the late Jurassic-early Cretaceous. It should be noted, however, that Wiens et al. [11] use a different date to calibrate the Amphisbaenia-Lacertidae split based on an older fossil from the early Cretaceous (98 Mya), Hodzhakulia magna [42,43]. This specimen consists only of incomplete maxillaries and dentaries and its purported amphisbaenian affinities have long been in doubt [44,45], making it problematic as a calibration point.

Overall, our dates for the origin of modern lacertids are much earlier than previous estimates, placing them in the late Paleocene, 58–56 Mya. Within the Lacertidae, the majority of divergences occur in the mid- to late Eocene after the Eremiadini split from their palearctic sister clade. The separation of the African clade into its Saharo-Eurasian and Ethiopian genera occurs shortly after, and they continue to diversify until well into the mid-Miocene, some 10 Mya. The relatively young ages of the African lineages are somewhat surprising given the high levels of species richness found in desert clades. Increased rates of speciation in desert lineages may be due to selection pressures experienced in extreme environments. Adaptations to xeric habitat favoring 'r-selected' strategies (e.g. reproducing and dying quickly) could promote a shift towards shortened generation times, thus accelerating diversification [7,46,47]. Unfortunately, very little is known about the ecology of desert lacertids, making it difficult to determine factors underlying their biogeographic patterns. However, recent studies indicate that physiological and life history variables, such as generation time, metabolic rate, body size and clutch size, influence mutation rates in terrestrial vertebrates [48,49], and may affect rates of molecular evolution in reptiles as well [50].

### Historical biogeography of Lacertidae

Most authors agree that lacertids originated in Europe, as indicated by the mainly European distribution of the basal Gallotinae [7]. According to our most reliable model (ULN), the majority of the lacertid radiation occurred in the mid-Eocene, 43–46 Mya. During that time, Europe was an archipelago of larger and smaller islands separated by shallow bodies of water [51]. The appearance of land bridges in the Eocene as well as increasing aridity are thought to have played an important role in terrestrial vertebrate migration, and evidence for faunal exchange between Europe and Africa can be seen in the fossil records of mammals and alligators [52,53]. A notable transition in fossil assemblages of squamate reptiles also occurs around the early Eocene in Europe, with large increases in diversity occurring at both the family and species level [[53] and references therein]. Unfortunately, the fossil record for African squamates during that time, particularly for small-bodied lizards, is poor [8] so that comparable estimates of lacertid diversity are unavailable. However, both the warming trend during the late Paleocene-early Eocene and low sea levels presumably made intercontinental dispersal feasible for a wide range of terrestrial vertebrates [53], possibly via land bridges or rafting.

**Figure 6:**
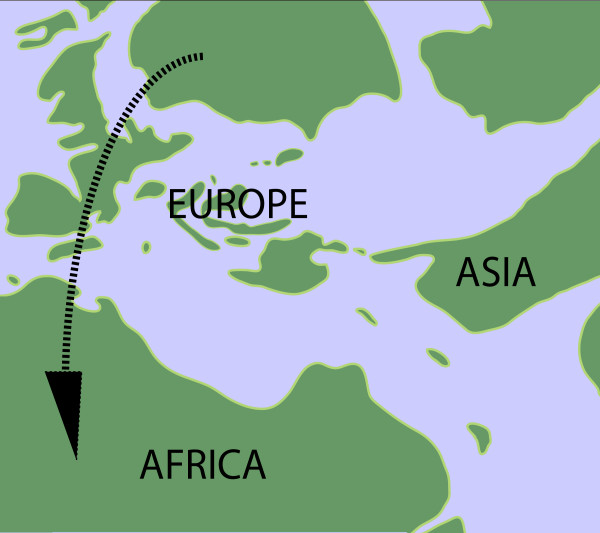
**Paleogeographic map of Europe and North Africa in the Late Eocene**. Arrow indicates possible lacertid migration route to Africa between southwestern Europe and northwestern Africa via small island chains.

One possible scenario is that lacertids entered North Africa at its northwestern edge via a chain of islands and diversified as they moved towards the southern tip of the continent (Figure 6; map after Popov et al. [54]). A primarily western migration for African lacertids is supported by modern biogeography, since the basal most taxa of both the European and African radiations are found along the western edges of the continents. The basal-most palearctic genus in our analysis (*Podarcis*; ULN, DM, CPP 50% consensus trees) occurs primarily in the western Mediterranean region and *Atlantolacerta andreanskyi*, which morphologically and genetically appears basal in the African radiation [7] is restricted to the Atlas Mountains in northern Africa. Taken together, these distributions indicate that southern Iberia and northwest Africa were important areas of divergence for modern lacertids. Similar pattern of dispersal have been hypothesized for other terrestrial fauna, where interchanges of mammals in the Cretaceous and Paleogene occurred along a discontinuous route between southwestern Europe and Africa [55]. Not until the mid-Miocene did a second, more stable land route between southeastern Europe and Asia form, permitting effective movement between the two landmasses [55].

Mayer and Benyr [56] and Arnold et al. [7] proposed the colonization of Africa by Lacertidae in the Miocene over the land bridge connecting Arabia and Africa, which remained up until the early Pliocene [57]. Although our dates for the initial radiation of African lacertids conflict with this hypothesis, this geological event could still have played an important role in the dispersal of certain members of the Saharo-Eurasian clade. Within that group, only *Adolfus *and *Holaspis *(the latter of which is absent in our analysis) are truly African in distribution, while the remaining genera are palearctic. Distributions of *Acanthodactylus*, *Mesalina*, and *Ophisops *in Africa are mainly restricted to the northern Atlantic coast, and the majority of their species, along with *Eremias*, are found in the Middle East and Asia. This pattern suggests that the Saharo-Eurasian lineage may have originated in Eurasia and only partially left the Palearctic, as opposed to secondarily recolonizing the Middle East and Asia from Africa. In fact, the land bridge could have been crossed in the other direction, with the ancestors of modern *Acanthodactylus*, *Mesalina*, and *Ophisops *entering Africa from Arabia once the continents established secondary contact.

An alternative colonization scenario is that the African lineage split from the Lacertini in Europe *prior *to migrating to Africa, and then only later radiated into its component lineages after reaching the African continent. Discovery of a fossil lacertid in Europe with African-like qualities would support this hypothesis. Interestingly, the Baltic amber lizard *Succinilacerta *[58] from mid-Eocene Poland was for some time assigned to the south African genus *Nucras *[59-61], suggesting that it resembles an African lacertid, at least superficially. Unfortunately, most of the diagnostic features separating the European and African clades, including features of the clavicle, tail, ulnar nerve and hemipenis, are not externally visible in preserved specimens [7]. Detailed investigation of this fossil, for example using X-ray Computed Tomography, could reveal internal structures assigning it to one of the modern clades. Other alternatives to fossil evidence may be found in additional taxon sampling for molecular studies. For example, inclusion of the basal African species *Atlantolacerta andreanskyi *in future molecular clock analyses could place a clearer temporal framework around the early evolutionary history of Eremiadini.

### Reliability of fossil calibrations

To test the reliability of the oldest squamate calibrations, the Teiidae-Amphisbaenia and the Amphisbaenia-Lacertidae splits, we alternately excluded each of them and compared their respective outcomes. Our results indicate that when all calibrations are combined, the teiid calibration does not have a significant effect on the estimated divergences for lacertids, since its exclusion only marginally alters the ages of the other splits (Figure 5). At the same time, when the teiid calibration is removed, its own divergence from lacertids/amphisbaenians becomes 17.4% older than the oldest-known teiid fossil, whereas removal of the amphisbaenian calibration causes the same split to be 64% older than the prior. Although this result may support the use of fewer calibrations, it should be noted that the use of only two fossil constraints led to unrealistic estimates, pushing the origin of Squamata well into the Permian.

With respect to future studies, we suggest that it may be preferable to constrain calibration points individually depending on the quality of the fossils themselves. For some clades, the quality of different fossils in terms of stratigraphic age or reliable phylogenetic position may be highly variable, with some being easier to constrain confidently based on prior knowledge than others. In such cases, the application of qualitative phylogenetic and stratigraphic criteria as suggested by Reisz & Müller [5,62] and Müller & Reisz [63] may be combined with exponential probability distributions, such that in case of a "good" fossil calibration, the soft bound spans the estimated temporal range in which the split must have occurred. Conversely, in cases of more questionable fossil dates, a 10% or 20% (or any other) upper bound may be applied.

## Conclusion

Estimation of evolutionary ages for crown clades such as the lizard family Lacertidae may be hampered by multiple sources of uncertainty, including unknown phylogenetic relationships, lack of an adequate fossil record, and variable evolutionary rates. These are not uncommon obstacles in molecular dating, however they must still be addressed within a statistical framework. Our results highlight the advantages of a Bayesian approach. The methods we describe allow incorporation of prior information in the form of multiple fossil calibrations, while allowing for statistical flexibility and the evaluation of alternative clock models using Bayes factors. We also support the use of a total evidence approach, in which all available molecular data is combined. Particularly when implemented with multiple calibrations in a Bayesian framework, the simultaneous analysis of multiple loci provides independent constraints on the evolutionary model, thereby avoiding potential biases associated with a single gene or genome [64]. Finally, we stress the importance of communication between paleontologists and molecular biologists in establishing suitable calibrations for more than just the major clades of Metazoa or Tetrapoda. Access to accurate information on divergence dates and paleontological material will allow biologists with diverse study systems to investigate topics such as evolutionary diversification, rates and patterns of morphological change, and historical biogeography at finer phylogenetic scales. In this regard, identifying groups needing additional study and developing plans to enable that study should be a top priority for paleontologists to position themselves as important contributors to the field of molecular dating.

## Authors' contributions

CAH collected genetic sequence data, performed the sequence alignment and drafted the manuscript. The original study was conceived by JM, who also participated in data interpretation and helped draft the manuscript. LH and DM designed the bioinformatics program used here and LH carried out all molecular clock analyses. All authors reviewed and approved the final manuscript.

## Supplementary Material

Additional file 1 **Lacertid clade ages**. Mean divergence dates, followed by error ranges, estimated under four different Bayesian molecular clock models using 10% and 20% prior probability distributions. Model abbreviations are Uncorrelated lognormal (ULN), Dirichlet Model (DM), Compound Poisson Process (CPP), and the strict molecular clock (MC). Additional analyses using the ULN model are ULN_1 (excluding the amphisbaenian and teiid calibrations), ULN_2 (excluding the amphisbaenian calibration), ULN_3 (excluding the teiid calibration), mtDNA partition, nDNA partition, and BEAST. Dates are given in millions of years. Nodes are numbered in correspondence to the tree in Figure 1 and calibrated nodes are denoted with an asterisk (*). Click here for file
