# Supplementary material for: Integration of Bayesian molecular clock methods and fossil-based soft bounds reveals early Cenozoic origin of African lacertid lizards
Source: BMC Evol Biol. 2009 Jul 1;9:151. doi: 10.1186/1471-2148-9-151 (PMC2719621; doi:10.1186/1471-2148-9-151)
Supplement: Additional file 1 — Lacertid clade ages. Mean divergence dates, followed by error ranges, estimated under four different Bayesian molecular clock models using 10% and 20% prior probability distributions. Model abbreviations are Uncorrelated lognormal (ULN), Dirichlet Model (DM), Compound Poisson Process (CPP), and the strict molecular clock (MC). Additional analyses using the ULN model are ULN_1 (excluding the amphisbaenian and teiid calibrations), ULN_2 (excluding the amphisbaenian calibration), ULN_3 (excluding the teiid calibration), mtDNA partition, nDNA partition, and BEAST. Dates are given in millions of years. Nodes are numbered in correspondence to the tree in Figure 1 and calibrated nodes are denoted with an asterisk (*). [file 1471-2148-9-151-S1.pdf]

Node	10% prior probability distribution										20% prior probability distribution		
	ULN	DM	CPP	MC	ULN_1	ULN_2	ULN_3	mtDNA	nDNA	BEAST	ULN	DM	CPP
1*	238.2 ± 10	236.9 ± 8.1	238.0 ± 10.8	231.6 ± 3.1	250.6 ± 18.1	239.0 ± 10.4	237.2 ± 8.6	235.8 ± 7.5	237.9 ± 9.4	233.5 ± 5.5	244.4 ± 15.3	246.2 ± 16.3	245.6 ± 16.0
2*	118 ± 4.5	118.9 ± 5.2	117.8 ± 5.2	124.9 ± 7.2	229.0 ± 25.3	127.2 ± 11.9	132.7 ± 25.9	116.9 ± 4.2	118.7 ± 5.5	117.3 ± 3.6	127.4 ± 11.5	128.5 ± 13.4	125.3 ± 9.7
3*	75.9 ± 7.4	73.6 ± 6.2	73.5 ± 5.0	72.2 ± 4.5	187.2 ± 27.8	105.3 ± 13.3	77.2 ± 8.2	79.9 ± 9.9	71.5 ± 6.3	68.5 ± 3.9	88.7 ± 11.5	89.4 ± 10.9	82.6 ± 8.1
4	58.3 ± 7.5	56.1 ± 6.3	43.3 ± 5.7	49.7 ± 3.2	136.6 ± 26.7	77.6 ± 13.2	60.0 ± 7.3	60.7 ± 9.5	55.0 ± 6.3	47.9 ± 4.9	67.1 ± 9.7	67.0 ± 9.3	51.8 ± 10.3
5	46.7 ± 6.1	44.1 ± 5.3	27.2 ± 5.3	39.5 ± 2.3	106.2 ± 19.9	60.1 ± 11.0	48.0 ± 6.6	48.8 ± 8	42.5 ± 5.9	36.1 ± 3.9	52.5 ± 7.5	52.0 ± 7.4	33.6 ± 8.4
6	43.2 ± 5.6	40.6 ± 5.1	24.7 ± 4.9	37.9 ± 2.1	96.8 ± 17.7	55.7 ± 10.1	44.3 ± 6.3	50.2 ± 7.6	39.1 ± 5.3	33.2 ± 3.7	48.5 ± 7.2	48.1 ± 6.5	30.4 ± 7.5
7	38.4 ± 5.1	35.9 ± 4.4	21.6 ± 4.5	35.0 ± 2.1	85.0 ± 16.4	49.0 ± 9.2	39.5 ± 5.8	43.8 ± 7.3	33.9 ± 5	29.4 3.4	43.2 ± 6.3	42.8 ± 5.8	26.8 ± 6.8
8	33.4 ± 5.2	31.4 ± 4.4	19.5 ± 4.1	29.2 ± 2.1	73.7 ± 14.9	42.3 ± 7.6	34.2 ± 5.9	36.4 ± 6.7	27.1 ± 4.9	25.1 ± 3.6	37.7 ± 5.8	37.0 ± 5.5	24.2 ± 6.0
9	18.5 ± 4.9	16.6 ± 4.3	10.9 ± 2.5	15.7 ± 1.7	39.9 ± 12.3	24.3 ± 7.2	19.0 ± 5.5	19.8 ± 5.8	14.7 ± 4.5	13.9 ± 3.4	20.0 ± 5.6	20.2 ± 5.1	13.5 ± 3.6
10	27.5 ± 5.2	25.5 ± 4.7	17.0 ± 3.7	24.9 ± 2.1	59.7 ± 14.0	34.8 ± 7.4	27.8 ± 5.8	30.8 ± 6.9	25.9 ± 5.1	20.4 ± 3.5	30.8 ± 5.5	30.3 ± 5.8	21.0 ± 5.4
11	35.1 ± 4.8	32.7 ± 4.4	19.5 ± 4.0	-	76.7 ± 15.6	44.5 ± 8.7	36.1 ± 5.6	42.5 ± 8.3	33.5 ± 5.1	26.9 ± 3.4	39.6 ± 6.3	39.0 ± 5.7	24.0 ± 6.2
12	30.4 ± 4.7	28.0 ± 3.9	17.1 ± 3.5	30.4 ± 2.0	65.0 ± 14.3	38.0 ± 7.6	31.2 ± 5.2	37.9 ± 8.7	27.3 ± 4.3	23.1 ± 3.3	34.2 ± 6.1	33.8 ± 5.1	21.1 ± 5.6
13	22.0 ± 4.6	20.9 ± 3.5	13.3 ± 2.8	24.6 ± 1.9	45.8 ± 12.5	27.7 ± 7.1	22.4 ± 4.9	26.2 ± 5.7	19.7 ± 4.1	16.8 ± 2.8	24.6 ± 5.4	24.2 ± 4.8	16.4 ± 4.3
14	40.2 ± 5.1	38.1 ± 5.1	22.8 ± 4.6	35.1 ± 2.1	89.7 ± 16.5	52.2 ± 9.3	41.4 ± 6.5	52.1 ± 7.8	33.7 ± 4.9	30.8 ± 3.6	45.6 ± 6.3	45.2 ± 6.0	27.8 ± 7.1
15	31.4 ± 4.8	29.7 ± 5.3	18.1 ± 3.9	31.0 ± 2.1	67.2 ± 15.4	40.3 ± 7.9	32.2 ± 5.7	43.0 ± 9.6	33.2 ± 5.1	24.4 ± 3.5	35.6 ± 6.1	35.2 ± 6.1	21.9 ± 5.8
16	10.3 ± 2.9	9.8 ± 3.0	4.9 ± 1.2	8.9 ± 1.1	23.2 ± 8.4	14.0 ± 5.2	10.4 ± 3.3	11.0 ± 3.4	9.2 ± 2.6	7.9 ± 1.9	11.7 ± 3.0	10.9 ± 2.9	5.7 ± 1.6
17	17.4 ± 3.4	15.5 ± 3.8	10.8 ± 2.2	14.6 ± 1.6	37.3 ± 10.5	21.6 ± 4.9	17.2 ± 3.8	25.2 ± 5.6	11.7 ± 3.5	13.8 ± 2.6	19.5 ± 4.5	18.6 ± 4.8	13.8 ± 3.6
18	22.0 ± 3.3	20.7 ± 4.0	15.1 ± 3.4	20.9 ± 1.6	48.8 ± 12.7	28.4 ± 6.4	22.8 ± 3.9	23.4 ± 4.3	19.9 ± 5.8	17.8 ± 2.6	25.7 ± 4.4	24.6 ± 4.1	19.2 ± 4.7
19*	5.9 ± 0.6	6.2 ± 0.8	6.0 ± 0.6	10.7 ± 1.0	5.9 ± 0.5	6.0 ± 0.6	5.9 ± 0.5	6.0 ± 0.6	5.7 ± 0.4	6.2 ± 0.6	8.4 ± 1.7	8.5 ± 1.6	9.2 ± 1.6
20	16.1 ± 3.2	-	-	-	33.1 ± 10.4	19.7 ± 5.1	16.2 ± 3.5	22.2 ± 4.7	19.6 ± 6.2	12.9 ± 2.5	17.9 ± 4.5	-	-
21	35.1 ± 5.6	33.3 ± 6.0	21.1 ± 4.5	26.3 ± 1.9	77.9 ± 18.9	45.8 ± 9.7	38.8 ± 6.3	36.3 ± 6.9	23.2 ± 5.1	26.8 ± 3.5	39.3 ± 6.5	38.9 ± 6.3	25.7 ± 6.7
22	19.7 ± 3.9	18.9 ± 4.5	13.9 ± 2.9	18.1 ± 1.6	44.2 ± 14.1	25.3 ± 6.5	20.5 ± 4.7	21.6 ± 6.2	12.4 ± 3.2	15.8 ± 3.0	22.6 ± 5.2	22.4 ± 5.1	17.2 ± 4.5
23	22.9 ± 6.7	21.0 ± 6.3	20.7 ± 3.4	16.9 ± 2.0	50.4 ± 17.3	27.3 ± 11.0	23.0 ± 7.8	19.6 ± 5.6	22.9 ± 7.7	16.7 ± 4.5	25.4 ± 7.6	22.1 ± 7.5	25.2 ± 7.3
24	32.3 ± 7.9	29.0 ± 7.8	26.6 ± 4.4	22.3 ± 2.3	74.5 ± 24.0	39.2 ± 13.2	31.4 ± 8.7	27.3 ± 6.8	33.0 ± 8.1	23.0 ± 5.3	35.1 ± 9.1	32.0 ± 9.8	32.8 ± 9.2
